# Development of Social Variation in Reproductive Schedules: A Study from an English Urban Area

**DOI:** 10.1371/journal.pone.0012690

**Published:** 2010-09-15

**Authors:** Daniel Nettle, Maria Cockerill

**Affiliations:** 1 Centre for Behaviour and Evolution, Institute of Neuroscience, Newcastle University, Newcastle, United Kingdom; 2 School Improvement Service, North Tyneside Council, Wallsend, United Kingdom; University of Otago, New Zealand

## Abstract

**Background:**

There is striking social variation in the timing of the onset of childbearing in contemporary England, with the mean age at first motherhood about 8 years earlier in the most deprived compared to the least deprived neighbourhoods. However, relatively little is known about how these social differences in reproductive schedule develop in childhood.

**Methodology/Principal Findings:**

We studied the development of differences in reproductive schedules, using a cross-sectional survey over 1000 school students aged 9–15 in the metropolitan borough of North Tyneside. Students from more deprived neighbourhoods had earlier ideal ages for parenthood than those from more affluent ones, and these differences were fully apparent by age 11. We found evidence consistent with three mechanisms playing a role in maintaining the socioeconomic gradient. These were: vertical intergenerational transmission (students whose own parents were younger at their birth wanted children younger); oblique intergenerational transmission (students in neighbourhoods where parents were younger in general wanted children earlier); and low parental investment (students who did not feel emotionally supported by their own parents wanted children at a younger age).

**Conclusions/Significance:**

Our results shed some light on the proximate factors which may be involved in maintaining early childbearing in disadvantaged communities. They help understand why educational initiatives aimed at adolescents tend to have no effect, whereas improving the well-being of poor families with young children may do so. Our results also suggest that there will be considerable intergenerational inertia in the response of reproductive schedules to changing socioecological conditions.

## Introduction

There is striking social variation in the timing of the onset of childbearing within contemporary affluent countries. For example, in England, mean age at first motherhood is about 8 years lower in the most deprived decile of neighbourhoods compared to the least deprived decile [1], and the occurrence of conceptions at a very young age is highly concentrated into the most deprived areas [2], [3], [4]. Elsewhere, we have discussed the ultimate reasons for the persistence of relatively early parenthood in more deprived neighbourhoods, which we argued to be the combination of relatively short healthy life expectancy with limited economic prospects. These combine to increase the costs and reduce the benefits of delaying childbearing [5]. Here, our focus is on the more proximate question of how people decide which age they feel to be the right one for the beginning of their childbearing.

It is clear that by the onset of adulthood, people have formed consciously-accessible intentions with regard to reproductive scheduling, which reflect their social context. For example, Nettle, Coall and Dickins [6] showed, using data from the National Child Development Study, that women's responses to the question ‘What do you think is an ideal age to have children?’, asked at age 16, varied with socioeconomic background. Moreover, the responses the women gave were quite a strong predictor of the timing of their actual subsequent childbearing. Thus, by the age of 16, young women have formed intuitions about the right age for parenthood, and these intuitions play out in their subsequent behaviour. This raises the first set of questions we wish to address in this study. How early in life are these social differences in intended reproductive schedule detectable? Would we find them in 14-year olds, or in children before puberty? Are they present in both sexes? And do they grow more marked with increasing age?

Our second set of questions concern *how* differences in intended reproductive schedule get formed. Influences are clearly being received during development in such a way as to cause a fairly stable setting of motivation towards early or delayed childbearing. What might these influences be? Several possibilities have been discussed. The first is vertical intergenerational transmission of some kind. That is, people whose own parents were young at the time of their birth tend to want to become parents young themselves. There is some evidence for intergenerational transmission of the timing of childbearing, even when continuities in the socioeconomic environment are controlled for [7], [8], although this is not found in all studies [9]. The second possibility is what has been called oblique intergenerational transmission [10]. That is, young people are influenced by the average age at which adults other than their parents who they encounter in their local environments have children.

A third possibility is calibration of reproductive intentions by particular cues from the local surroundings. Wilson and Daly [11] demonstrated a close relationship between local homicide rates and rates of teen pregnancy in Chicago neighbourhoods, and suggested that specific psychological mechanisms respond to experiences of mortality going on in the immediate surroundings with an acceleration of reproductive motivation, an idea for which there are various sources of supporting evidence [12], [13], [14], [15]. The argument can be extended to encompass social disorder, agonistic interactions, or other cues that the environment is generally unsafe [16]. The fourth possibility is that young people are sensitive to cues coming from the behaviour of caregivers. In more deprived areas, parents tend to invest less care in each child [1], [17], and low parental investment has been argued to trigger an acceleration of developmental schedule in the child, at both the physical and psychological levels [18]. There are many studies showing that such markers as father absence, separation from parents, limited breastfeeding, or poor relationships with parents are associated with such markers of accelerated maturational schedule as early menarche [19], [20], [21], [22], [23], [24], early sexual activity [25], [26], or early childbearing [8], [23], [27].

Thus, to summarise, young people in more deprived areas may wish to have babies sooner (1) because their own parents were younger, (2) because other adults they see having children are younger, (3) because they have formed the perception that their environment is unsafe, or (4) because they have received less parental investment than their peers from more affluent areas. Our goal was to test for evidence consistent with any or all of these mechanisms operating to maintain preferences for earlier parenthood in more deprived areas.

We also tested a more proximal psychological hypothesis about how these influences could lead to earlier ideal ages for parenthood, namely that they do so by causing young people to believe that their lives are going to be shorter. Female life expectancy is, at the global level, the strongest predictor of age at first parenthood [5], [28]. A number of studies have shown that women who become mothers early within affluent populations have an expectation that their lives will be relatively short [29], [30]. Studies have also shown that subjective life expectancy is lower in people of lower socio-economic position [31], and lower in people who experience low levels of family support in childhood [23], [32], [33]. Thus, subjective life expectancy could be an important intermediate psychological state between childhood developmental influences and the formation of reproductive goals [34].

In this study, then, we examine the development of intended reproductive schedules in a large sample of school students within an urban area in Northern England, the metropolitan borough of North Tyneside. This is a socioeconomically mixed area, where material conditions vary markedly over the space of a few kilometres. We will first examine whether there are differences in intended reproductive schedule according to objective indicators of local socioeconomic conditions in the neighbourhood the respondent lives in. We will then endeavour to establish whether these differences are mediated by any or all of the four sets of developmental influences listed above. Finally, we examine the role of subjective life expectancy as a proximal psychological correlate of intended reproductive schedule.

## Methods

### Study area

North Tyneside is a metropolitan borough forming part of the Tyne and Wear conurbation in Northeast England. It occupies around 80 km^2^ and had a population of 191,659 including 36,779 people under 16 at the 2001 Census. In the 2007 national indices of economic deprivation, North Tyneside is ranked the 102^nd^ most deprived of 354 local authority areas [35]. However, this masks marked internal heterogeneity. The borough has areas of extreme deprivation, with the Chirton ward in the most deprived 1% of all English electoral wards, and areas of relative affluence, with the St. Mary's ward in the 90% percentile of deprivation by the same measure [36].

### Sample

We worked with an opportunity sample of eight local schools who were partners in a broader programme of research and intervention aimed at improving young people's psychological wellbeing. The number and age-profile of the sample varied from school to school according to the demands of the school timetable, the priorities of teachers, and other factors which are essentially random with respect to the objectives of this study (*n* per school 20-378). However, each school provided a cross-section of students of the particular ages they chose to work with, by having whole classes participate, and most schools provided several age groups. The total sample of 1149 students (596 female) was made up of 409 9–11 year olds, 396 12–13 year olds, and 346 14–15 year olds. The schools covered a broad spectrum of the borough, although the different neighbourhoods were not sampled proportionally to their populations.

### Survey data

Students completed an anonymous online survey in their school classrooms, during the school day. All respondents worked individually at a computer. The chief outcome variable was the response to the question ‘What do you think is an ideal age to have children?’ This was answered by moving a draggable visual slider along a scale running from 0 to 50 with guide lines at 10-year intervals, with the initial position at 0 and a numerical readout of the current position of the slider by the side. Respondents gave their own ages, and those of their mothers and fathers, in free text responses, and we used these to calculate the ages of both of their parents at the time of their births. Since maternal and paternal ages at respondent's birth are highly correlated with one another (r_848_ = 0.70, p<0.05), we calculated the mean of the respondent's maternal and paternal ages as our ‘own parents' age’ variable.

Our measure of the perception of the safety of the environment was the question ‘On a scale of 1–100, how safe do you think your neighbourhood is?’, answered using a visual slider. Responses to this item correlated substantially with responses to how much people in the neighbourhood could be trusted (r_1032_ = 0.69, p<0.05), and how much crime the respondent felt there was in the neighbourhood (r_1061_ = −0.46, p<0.05). We do not consider these other items further here.

For parental investment, we administered a short family support scale modelled on the family stress scale of Mikach and Bailey [37], which has been used elsewhere to test hypotheses about the relationship of parental investment and life history strategy [23]. The 5 items, which are answered on a 7-point scale of ‘Strongly Disagree’ to ‘Strongly Agree’ are: ‘My father is always there when I need him’, ‘I want to raise my children in the way my parents raised me’, ‘My mother is always there when I need her’, ‘I do many activities with my family’, and ‘My parents always seem to care about what I am doing’. Scale reliability was high (α = 0.78), and we summed items to produce an overall score (higher score indicates a more supportive family). As a validity check, this score varies with the composition of the respondent's residential family unit (F_4,970_ = 36.60, p<0.05), being highest in the respondents who live with both biological parents (M 29.36), followed by those who live with a parent plus step-parent (M 26.13), those who live in some other composition (M 24.71), those who live with a lone parent (M 24.64), and finally those who live with neither parent (M 21.10).

The area of the borough which the respondent came from was established by a free text response to the question ‘Which area of North Tyneside do you live in?’ We matched this response to one of the 20 electoral wards of which the borough is composed (2000 administrative boundaries). 104 students could not be assigned a ward because although they attended a North Tyneside school, they resided outside borough boundaries, or else their responses were insufficiently specific. For each ward, we obtained the Index of Multiple Deprivation (IMD) for 2000 [the most recent available data for these geographical units, 36]. The IMD is a composite index of neighbourhood-level socioeconomic hardship which takes into consideration indicators of in the domains of income, employment, health, education, housing, and access to services (higher scores indicate more deprived neighbourhoods; range of IMD scores 6.73–70.85).

The sample contained some respondents from 17 of the 20 electoral wards in the borough, but several wards had very few cases (9 wards with fewer than 50). Thus, we amalgamated wards in such a way that no area had fewer than 50 respondents, using the principles (a) that wards were only amalgamated with adjacent wards; and (b) no wards whose IMD scores differed by more than 5 were amalgamated. This procedure produced a final set of 8 large neighbourhoods, each containing 65–233 respondents. The IMD for these composite neighbourhoods was calculated as the mean of the IMDs of the constituent electoral wards, weighted by the number of respondents that each constituent ward supplied. We also calculated the mean across respondents in each neighbourhood of the parental age for that neighbourhood, which we used as the ‘neighbourhood parents' age’ variable, to test for oblique transmission.

### Analysis

Since our sample consists of respondents who are clustered within neighbourhoods, and both respondent-level and neighbourhood-level influences of ideal age for parenthood could be at work, we used multilevel regression modelling with MLwiN [38], treating respondents as the level 1 units, and neighbourhoods as the level 2 units within which respondents are nested. Individual parameters were considered statistically significant if their 95% confidence intervals did not include zero. A model was considered a significant improvement over a simpler model based on the change in −2loglikelihood, which under the null hypothesis follows a χ*^2^* distribution with *k* degrees of freedom, where *k* is the number of additional parameters in the more elaborate model [39].

We first built a base model with just age group and sex as level 1 predictors, and then added neighbourhood IMD, a level 2 predictor, to establish whether neighbourhood-level deprivation was associated with ideal age for parenthood. We then tested whether any effect of neighbourhood IMD was explained by differences in parental age, parental investment, or perception of neighbourhood safety. To do this, we first established that these variables did in fact covary with neighbourhood IMD, and then added each of them in turn to the regression model. If the effect of neighbourhood IMD then ceases to be significantly different from zero, then it is completely mediated by these additional variables. Finally, we added subjective life expectancy to the best-fitting overall model, to establish whether this mediated any of the relationships observed, testing mediation effects with the Sobel test [40].

### Ethics statement

This study was approved by Psychology Ethics Committee at Newcastle University, and carried out with the agreement of all participating schools and of North Tyneside council. Participants completed the survey during class time, but were free to not submit responses or to omit questions. The survey software indicated that by clicking the final submit box, participants would be consenting to have their responses analyzed as part of a research study. No individually identifying personal information were asked for or available to the researchers.

## Results

**Figure 1:**
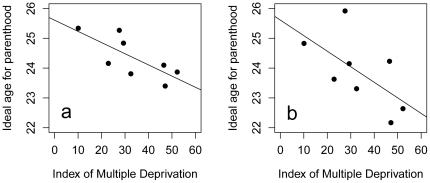
Marginal mean for each neighbourhood of ideal age for parenthood, adjusted for age group and sex, against the Index of Multiple Deprivation of that neighbourhood (a) for the whole sample, (b) for the 9–11 year olds only.

Table 1 presents descriptive statistics for the main variables included in the study, for the 1046 responses which could be assigned to a neighbourhood. We first fitted a multilevel model with respondents nested within neighbourhoods, and age group and sex as the predictor variables (model 1 in table 2). This revealed a significant effect of age group, explained by 14–15 year olds having higher ideal ages for parenthood than the other two age groups (means, 9–11: 24.21, 12–13: 23.89, 14–15: 25.17). There was no significant sex difference in ideal age for parenthood (means, male: 24.31, female: 24.42). The overwhelming majority of the variation (99.2%) was at the between-respondent level rather than between neighbourhoods. Nonetheless, when we added neighbourhood IMD as a level 2 predictor, there was a significant effect, with a negative parameter estimate indicating higher neighbourhood IMD was associated with lower ideal ages for parenthood (model 2 in table 2). To visualise this effect, figure 1a plots the marginal mean ideal age for parenthood, adjusted for sex and age group, for each neighbourhood, against that neighbourhood's IMD score. As the figure shows, the more deprived neighbourhoods generally have lower ideal ages for parenthood. When interaction terms were added to model 2, there were no significant interactions between neighbourhood IMD and sex (B = 0.01, s.e. = 0.02, n.s.) or age group (IMD*12–13 B = 0.01, s.e. = 0.03; IMD*14–15 B = 0.04, s.e. = 0.04, n.s.). This suggests that the IMD-ideal age for parenthood relationship is not restricted to the older respondents, and figure 1b confirms this by plotting the association between neighbourhood IMD and ideal age for parenthood for the 9–11 year olds only.

**Table 1:** Descriptive statistics (mean and standard deviation, or list of values, as appropriate) for the main variables in the study.

Variable	Descriptives
Ideal age for parenthood (years)	24.38 (4.84)
Own mother's age (years)	27.99 (5.64)
Own father's age (years)	30.48 (5.98)
Own parents' age (years)	29.36 (5.26)
Family support	27.82 (5.81)
Perceived neighbourhood safety	66.94 (27.17)
Neighbourhood IMD	10.07, 22.90, 27.57, 29.35, 32.48, 46.52, 47.12, 52.24
Neighbourhood parents' age	30.72, 28.67, 31.53, 29.15, 28.67, 28.70, 27.36, 27.67
Subjective life expectancy	86.60 (16.48)

Maternal and paternal ages are at the time of the respondent's birth, not the time of survey.

**Table 2:** Summary of multilevel regression models with ideal age for parenthood as the outcome variable.

Variable	Model 1	Model 2	Model 3	Model 4	Model 5	Model 6	Model 7	Model 8
Agegroup 12–13_1_	−0.45 (0.36)	−0.53 (0.36)	−0.45 (0.39)	−0.56 (0.36)	−0.32 (0.36)	−0.36 (0.34)	−0.29 (0.39)	−0.31 (0.39)
Agegroup 14–15_1_	1.02* (0.39)	1.00* (0.38)	1.44* (0.42)	1.10* (0.38)	0.93* (0.38)	1.32* (0.37)	2.02* (0.42)	1.96* (0.43)
Sex_1_	0.14 (0.30)	0.12 (0.30)	0.05 (0.33)	0.13 (0.30)	0.14 (0.30)	0.01 (0.28)	−0.02 (0.33)	0.00 (0.32)
Neighbourhood IMD_2_		−0.04* (0.02)	−0.02 (0.02)	−0.01 (0.02)	−0.03* (0.01)	−0.03 (0.02)	0.00 (0.04)	0.00 (0.02)
Own parent age_1_			0.13* (0.03)				0.12* (0.03)	0.12* (0.03)
Neighbourhood parent age_2_				0.39* (0.14)			0.47* (0.16)	0.46* (0.16)
Neighbourhood safety_1_					0.01 (0.01)		−0.01 (0.01)	−0.01 (0.01)
Family support_1_						0.12* (0.03)	0.12* (0.03)	0.11* (0.03)
Subjective life expectancy_1_								0.01 (0.01)
−2loglikelihood	6074.58	6069.94	4479.37	6062.96	5794.38	5632.95	4186.14	4169.77
Pseudo R^2^	1.4%	1.6%	15.6%	2.0%	5.0%	17.4%	19.9%	19.8%

Predictor variables are subscripted with a 1 if they are at the level of the individual respondent, and a 2 if they are at the neighbourhood level. Reference categories are ‘9–11’ for age group and ‘male’ for sex. Values given are parameter estimates with their standard errors in parentheses. An asterisk indicates that the individual parameter estimate differs from 0 at the 5% level. The pseudo R^2^ is the proportion of individual-level (level 1) error variance explained by this model compared to an intercept-only model.

**Figure 2:**
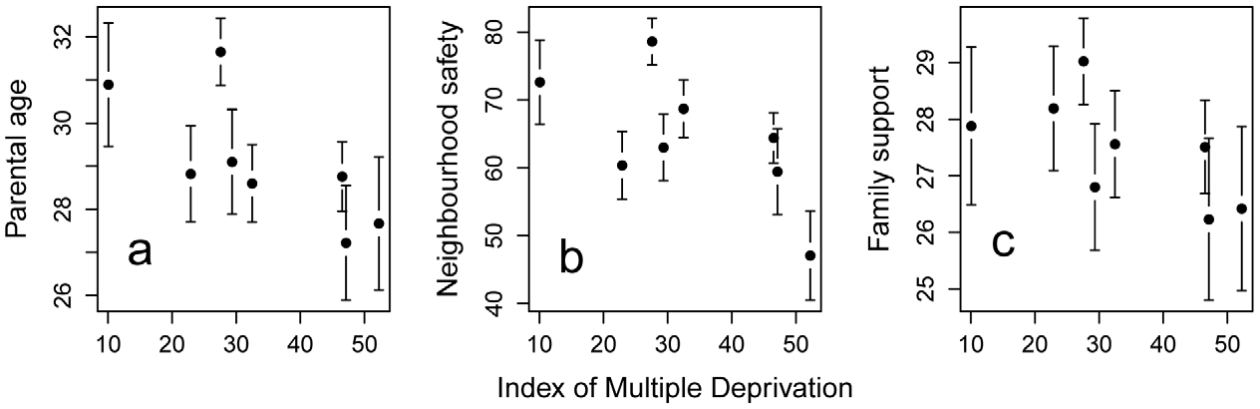
Mean for each neighbourhood of (a) respondent's parents' age at the time of their birth; (b) perceived neighbourhood safety; and (c) family support, against the Index of Multiple Deprivation for the neighbourhood. Error bars represent 95% confidence intervals for the mean.

**Figure 3:**
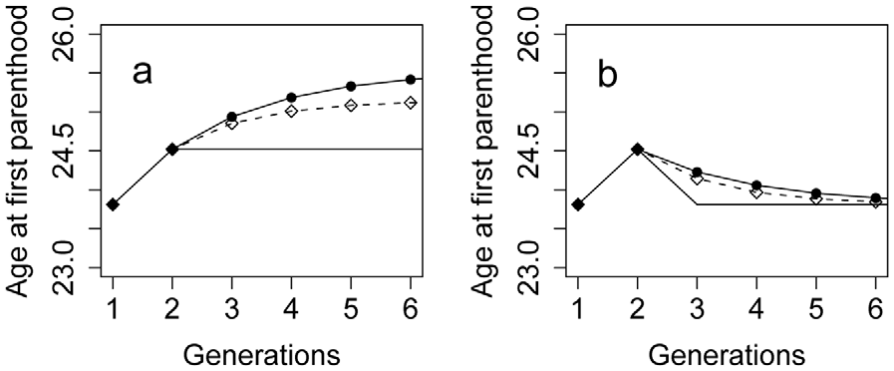
Predicted responses to a hypothetical intervention in the most deprived neighbourhood we studied whose effect is to raise family support by one standard deviation (equivalent to an increase in score of 5.91). We assume that changes in ideal age for parenthood lead to older ages at actual parenthood, and that these feed iteratively into the formation of the intended reproductive schedule of the next generation by cultural transmission. Parameter values used are drawn from Model 7 in table 2. (a) The intervention is implemented during the childhood of generation 1, and remains in place permanently. The plain line indicates the direct effect of the intervention alone. The line with solid circles represents its total effect, assuming that both the vertical and oblique intergenerational influences are cultural ones. The line with open diamonds represents its predicted total effect if the oblique effect is a cultural one, with the vertical intergenerational transmission being genetic and so not responding to the change in the mean parental age. (a) The intervention is implemented during the childhood of generation 1 for one generation only, and levels of family support return immediately to baseline. Again, the plain line is the direct effect of the intervention alone, the line with solid circles assumes that both the vertical and oblique effects are cultural ones, and the line with open diamonds assumes that only the oblique effect is a cultural one.

Our possible developmental mediators of the neighbourhood differences, namely parental age, perceived neighbourhood safety, and family support, did all show some variation across neighbourhoods, and some patterning with neighbourhood deprivation (figure 2). Own parents' age and neighbourhood safety were significantly negatively associated with neighbourhood IMD (midparent age: B = −0.07, s.e. = 0.03, p<0.05; neighbourhood safety: B = −0.44, s.e. = 0.18, p<0.05), whilst the relationship between family support and neighbourhood IMD, though negative, did not reach statistical significance (B = −0.05, s.e. = 0.03, p = 0.08; figure 3). Thus, it is possible that inter-neighbourhood differences in these variables mediate the inter-neighbourhood differences in ideal age for parenthood.

When own parents' age, neighbourhood parents' age, perceived neighbourhood safety, and family support are entered in turn into the model with age group, sex and neighbourhood IMD, each of them significantly improves the model fit (models 3–6 in table 2). The effects are all in the predicted direction, with younger parents associated with younger ideal ages for parenthood, neighbourhoods perceived as more safe associated with later ideal ages for parenthood, and higher family support associated with later ideal age for parenthood, although the parameter estimate for neighbourhood safety is not significantly different from zero. If either of the parental age variables, or family support, is entered into the model, the effect of neighbourhood IMD is no longer significant (models 3,4,6). Thus, the neighbourhood gradient in ideal age for parenthood is completely mediated by neighbourhood differences in own parents' age, neighbourhood average parents' age, and family support. The best-fitting model overall contains all four of the additional predictor variables (model 7 in table 2). In this model, the mutually-adjusted parameter estimates for own parent's age, neighbourhood parents' age, and family support are all significantly different from zero. We also tested interaction effects between each of the predictors and sex, and each of the predictors and age group. In no case were there any significant interactions (data not shown).

To examine whether the effect of own parents' age is driven by the parent of one sex more strongly than the other, we performed partial correlation analyses between ideal age for parenthood and own mother's age controlling for own father's age, and own father's age controlling for own mother's age. For the boys, neither correlation is significant (controlling for own father's age: r_322_ = 0.08, n.s.; controlling for own mother's age: r_322_ = 0.06, n.s.). For the girls, however, the correlation controlling for own father's age was significant (r_439_ = 0.19, p<0.05), whilst that controlling for mother's age was not (r_439_ = −0.05, n.s.). Thus, for the boys, neither parent is more influential than the other, whilst for the girls, there is some evidence of specific mother-to-daughter transmission.

Finally, we tested for proximal psychological mediation by subjective life expectancy. The precondition for mediation is that the candidate mediator variable is associated with both the predictor and the outcome variables [41]. In a simple correlation analysis, subjective life expectancy was weakly associated with the outcome, ideal age for parenthood (r_1019_ = 0.07, p<0.05), and also with family support (r_978_ = 0.10, p<0.05) and neighbourhood safety (r_982_ = 0.10, p<0.05). Since the association between neighbourhood safety and ideal age for parenthood was not significant, there was only one mediation relationship to test, namely that of family support to ideal age for parenthood by subjective life expectancy. When subjective life expectancy is added to the model (table 2, model 8), although the model fit is significantly improved, the parameter estimate for family support is hardly changed (0.11 vs. 0.12.). A Sobel test reveals no significant mediation effect (Sobel *z* = 0.74, n.s.).

## Discussion

Our results show that the socioeconomic differences in target age for parenthood which we know are present in British adults [42] and adolescents [6], are already present in childhood. Respondents from the most deprived neighbourhoods stated ideal ages for parenthood that were several years younger than those from the more affluent ones, and this pattern was no less marked in the 9–11 year olds than in the older children (figure 1). The pattern was not restricted to one sex or the other. In terms of the mechanisms sustaining these social differences in intended reproductive schedule, we found evidence consistent with both vertical and oblique intergenerational transmission, and also with a role for parental investment, but no evidence suggesting an effect of perceived neighbourhood safety.

Individuals' ideal ages for parenthood were associated with their own parents' age at their birth, and also with the average age of adults in their neighbourhood at the birth of children. The finding of vertical intergenerational transmission replicates some previous observations in the literature on early childbearing [7]. Of course, these data are uninformative with regards to whether any such transmission is genetic, or operates through social learning. Both are possible [for evidence of genetic effects], [ see 43,on the plausibility of social learning, see 44], but different study designs from the one employed here would be required to tease them apart. The data suggest that, for a girl, it is the age of her mother which is especially influential in forming her ideal age for parenthood, whereas for a boy, neither parent is more influential than the other. Our study is novel in suggesting that oblique intergenerational transmission may also be important; children from neighbourhoods where parents are generally younger want to become younger parents themselves, even once the age of their own parents is adjusted for. Oblique transmission, of course, can only be via learning, and not genetic, and it could provide an explanation for why previous studies of teenage pregnancy or childbearing have sometimes found predictive effects of area-level socioeconomic variables above and beyond the effects of family-level ones [2], [3].

We also found clear evidence that receiving lower parental investment or having poorer relationships with parents is associated with a ‘speeding up’ of reproductive strategy, indicated in our case by earlier ideal ages for parenthood. This accords with a large number of findings showing associations between measures of reproductive schedule and measures of parental investment of parent-child relationships [see for example 8], [18], [19], [20], [23], [24], [25], [26], [27]. However, our study is relatively unusual in using consciously-stated reproductive goals as an outcome measure, rather than a physical development measure such as age at menarche, or a behavioural one such as age at first conception. Our study is also relatively unusual in that it shows that the parental investment-reproductive schedule relationship is the same in boys as in girls. Since so many previous studies have used age at menarche as their outcome, they have only been able to include girls [see 45 for an exception], whereas our measure allows both sexes to be studied. Indeed, one of the striking features of our data is that there were few differences between boys and girls, either in their baseline ideal ages for parenthood, or in the effects of the predictors.

We found that subjective life expectancy was weakly associated with family support, with young people who experienced low family support feeling that they would live less long. This is consistent with the findings of a number of other studies [23], [32], [33]. Subjective life expectancy was also weakly associated with ideal age for parenthood, with young people who felt they would live longer being prepared to delay their childbearing longer, again consistent with both theoretical expectation and the findings of a number of other studies [23], [29], [30], [34]. However, the association between subjective life expectancy and ideal age for parenthood became nonsignificant in a fully-adjusted model, and subjective life expectancy did not mediate the relationship between family support and ideal age for parenthood.

The data presented here have a number of limitations which make conclusions about causal pathways necessarily tentative. First, our outcome measure is only a stated ideal for age at parenthood; we have not followed our cohort to examine childbearing itself. It could be the case that stated ideals in childhood, and actual behaviour, have quite different predictors. However, our previous work with the National Child Development Study cohort suggests that this is not the case. Ideal ages for parenthood, stated at 16, are a surprisingly good predictor of actual subsequent behaviour [6], and moreover, actual age at first childbearing in that cohort is predicted by parents' age and parental investment in a very similar way to stated ideal age in this study [8]. This suggests that examining young people's stated intuitions about when is a good age to have a family is a worthwhile exercise, which has implications for real-world decisions. Second, our data are cross-sectional rather than longitudinal. This is a particular limitation in the case of parental investment associations. Ideally, one would have objective measures of parental investment gathered in early childhood, and outcome measures gathered later [as in 8]. Instead, in our study, both independent and dependent variables were gathered simultaneously and from the same source, raising the possibility of the associations being confounded by current mood or other response biases. Third, and more generally, associations between ideal age for parenthood and other factors may reflect the common effects of some unmeasured variables rather than any direct causal relationship. Finally, even all of our predictor variables entered together accounted for only a minority of the variation in stated ideal age for parenthood.

These limitations duly noted, we do feel that our findings are at least suggestive for helping understand the persistence of early childbearing in deprived areas. Teenage childbearing has received considerable social policy attention and government intervention in recent years, though the basis for viewing it is a problem requiring intervention is questionable [46], [47]. Teenage childbearing occurs overwhelmingly in the most deprived neighbourhoods, and is basically a side-effect of the fact that the whole distribution of age at first childbearing is shifted younger in these neighbourhoods. In our earlier work, we suggested that those interested in influencing the rate of early childbearing need to pay attention to the ultimate causes – short life expectancies and poor economic prospects – which favour generally early childbearing in these communities [1] (see also [48] for this argument in the context of health interventions more generally). Here, our data also suggest that understanding the mechanisms by which reproductive schedules are formed is important for predicting the effects of interventions. The neighbourhood differences in ideal ages for parenthood are already well established by ages 9–11, which concurs with recent evidence that, though interventions in adolescence tend to have no effect [49], those acting earlier in childhood may do so [50]. Moreover, the data we have presented suggest that there are powerful formative influences lying behind decisions about when to have babies; influences from one's parents, from one's surrounding community, and from the level of parental input one has received. It should not surprise us, then, that early childbearing is resistant to change by simple information-giving. In particular, where there is intergenerational cultural transmission, as seems to be the case here, there can be behavioural inertia lasting several generations, even if the ecological conditions which gave rise to the behaviour change [10], [51].

To illustrate this point, in figure 3, we use the empirically-observed relationships in our data to model the predicted response to a hypothetical intervention in the most deprived of the neighbourhoods we studied, whose effect is to raise the investment of parents in their young children by one standard deviation, either by reducing the economic strains on these families, or by some other means. We are assuming here that the cross-sectional associations in our data reflect direct causal relationships, an assumption which may not be justified, and so our scenario is to be treated as an illustration rather than a precise prediction. Let us assume that the hypothesized intervention is wholly effective, and that it remains in place permanently after having been begun (figure 3a). In the second generation of residents, age at first parenthood is predicted to be later than in the first because of the relationship between parental investment and intended reproductive schedule (we assume here that a change in intended reproductive schedule turns into a corresponding change in actual reproductive behaviour). In the third generation, the predicted effect is even larger because now as well as the increased parental investment, children are exposed to older parents in the neighbourhood (because of the change which occurred in generation two), and so their reproductive schedules are further delayed by vertical and oblique intergenerational transmission. Thus, the effectiveness of the intervention is magnified by the intergenerational transmission, and in fact, it would not be expected to have its asymptotic effect for several generations, even though the intervention itself does not change after it is first implemented. This conclusion is not much changed if we assume, conservatively, that the vertical intergenerational effect is entirely genetic, and only the oblique effect is based on learning (figure 3a, lower line).

Now consider a one-off intervention that raises the level of parental investment in children for one generation, but is not continued, and the level of parental investment returns immediately to baseline. Our model predicts that although the intervention will have no direct effect on the children of the third generation, it has an indirect effect via exposing them culturally to older parents. Thus, the total impact of the intervention would not have dissipated for several generations (figure 3b). Again, this conclusion is not much affected by conservatively assuming that only the oblique and not the vertical effect is a cultural one (figure 3b, lower line).

These predictions, speculative as they are, demonstrate several things. One is the potential potency of community interventions which raise the level of parental investment in children in terms of making an impact on early childbearing. This concurs with reports in the literature of programmes which work to improve relationships between parents and very young children having a knock-on effect on teenage pregnancy rates many years later when those children have become adolescents [50]. Second, it is clear that the extensive theoretical literature on vertical and oblique cultural transmission and its consequences [10], [44] is relevant to understanding the social dynamics of reproductive behaviour. This means that this body of theory could be brought to bear to help understand how social variation in reproductive behaviour is maintained, how it respond to changes in the environmental context, and how and over what timescale interventions should be evaluated.
